# Electrophysiological Measures of Visual Working Memory in Social Anxiety

**DOI:** 10.3389/fnbeh.2020.00049

**Published:** 2020-04-17

**Authors:** Jing Yuan, Ningning Mao, Rongrong Chen, Qin Zhang, Lixia Cui

**Affiliations:** ^1^Beijing Key Laboratory of Learning and Cognition and School of Psychology, Capital Normal University, Beijing, China; ^2^School of Teacher Education, Hebei Normal University for Nationalities, Chengde, China

**Keywords:** social anxiety, visual working memory, event-related potentials, attentional control, CDA

## Abstract

Socially anxious individuals are very sensitive to threatening information in the environment, so visual working memory (VWM) is of great significance for them. However, the influence of social anxiety on VWM is unclear. In the present study, we aimed to investigate the VWM in individuals with social anxiety using electrophysiological techniques. Event-related potentials (ERPs) of high socially anxious (HSA) individuals and low socially anxious (LSA) individuals were recorded during a change-detection task with two memory conditions (two and four items). Electrophysiological results indicated that compared with the LSA individuals, the HSA individuals had significantly more active contralateral delay activity (CDA) in condition of memorizing four items. However, there was no significant difference between the HSA and LSA groups in response accuracy in the conditions memorizing two and four items. From the electrophysiological results, individuals with high social anxiety could maintain more information in VWM. However, maybe anxiety consumes the available cognitive resources to compensate for the supposed to be impaired effective performance, so that individuals with high social anxiety perform the same as individuals with low social anxiety in terms of behavioral outcomes.

## Introduction

Social anxiety is an aversive emotional and motivational state characterized by an avoidance of social situations and a fear of negative evaluation (D’Avanzato and Dalrymple, 2016). Individuals with social anxiety usually express attentional bias when they process threated information (Chen et al., 2016; Wieser et al., 2018), which is thought to play an important role in the maintenance and development of social anxiety disorders (Rapee and Heimberg, 1997; Heimberg et al., 2010).

Compared with nonemotional stimuli, social-related threat will capture more attention of individuals with social anxiety (Grafton and MacLeod, 2016; Lazarov et al., 2016). Eysenck et al. (2007) proposed that anxiety impaired attentional control processes by interfering with the balance between stimulus-driven and goal-directed attentional systems. Specifically, anxiety impairs two functions of attentional control, that is, inhibition and shifting, which leads to a decreased influence of the goal-directed attentional system and an increased influence of the stimulus-driven attentional system. Liang (2018) specified that the attentional control deficit in social anxiety was mainly inhibition rather than shifting. It follows that inhibitory deficit may lead socially anxious individuals to invest excessive resources in processing task-irrelevant threat information (Dodd et al., 2017). However, there is also evidence that individuals with social anxiety perform as well on many cognitive tasks as individuals without social anxiety, even on task related to emotion (Waechter et al., 2018). In the common view, individuals with more attentional resources can process more stimuli than individuals with those fewer attentional resources. Under this line of reasoning, anxious individuals should have more cognitive resources so that they can process both task-relevant stimuli and task-irrelevant distractors and, due to inhibitory defect, allocating attention to the latter.

Visual working memory (VWM) is a limited resource and is closely related with attention (Awh et al., 2006; Chun, 2011). They share the same capacity, share the same control process, and share the same content (Olivers, 2008), although VWM and attention is not one simple unity (Tas et al., 2016). The relationship between VWM and attention is strongly dependent on the demands placed on perceptual and memorial selection. When the source of attention search is the memorial demands, the overlap of VWM and attention will be observed (Woodman et al., 2007). In this study, we wanted to explore the cognitive resources of individuals with social anxiety through VWM.

Studies have reported that the VWM capacity stored up to three to four visual objects (Awh et al., 2007; Fukuda and Vogel, 2009). However, there is a debate on the unit of VWM, object, variable or more widely (Wheeler and Treisman, 2002; Zhang and Luck, 2008; Anderson et al., 2011; Huang, 2020). Contralateral delay activity (CDA) is a negative slow-wave sensitive to reflect the amount of information held in VWM (Vogel and Machizawa, 2004; Drew et al., 2006; Feldmann-Wüstefeld et al., 2018), which can dissociate accuracy and the maintained representations in VWM (McCollough et al., 2007). The change-detection task is usually employed to measure CDA (Luria et al., 2016; Adam et al., 2018). In the change-detection task, a memory array containing colored squares presents in the left and right visual hemifields and is preceded by cues that specify which side of the memory array has to be retained for subsequent comparison with a test array. Event-related potentials (ERPs) recording the maintenance phase revealed sustained enhanced negativity at electrodes contralateral to the to-be-remembered display side.

The study on the role of social anxiety on VWM is fewer. Meconi et al. (2014) asked participants to perform a change-detection task while their CDAs were recorded. The memory array consisted of two faces presented in each visual hemifield, preceded by an arrow cue indicating to the side of the to-be-memorized face. Participants were required to examine the same precued side of the test array for a possible change in the identity of the face. The result showed that individuals with high anxiety (state and social anxiety) levels had larger CDA than individuals with low anxiety levels, which suggested that high anxious individuals could memorize greater detail of untrustworthy faces compared with low anxious individuals. We want to know whether this conclusion can be generalized to nonemotional stimuli, that is, whether individuals with high social anxiety (HSA) have higher VWM capacity in non-emotional stimuli compared to individuals with low social anxiety (LSA).

It is greatly important for understanding the mechanism of anxiety to explore attention for nonemotional processing in individuals with social anxiety. If HSA individuals have high VWM capacity in nonemotional stimuli than LSA individuals, it will provide indirect support to the opinion that HSA individuals have problems in attentional control (Eysenck et al., 2007). Specifically, due to inhibition deficits (Derakshan et al., 2009; Calvo et al., 2012), anxious people with more VWM resources perform the same as (or worse than) low-anxiety people on the cognitive tasks who would have performed better.

The current study focused on the VWM of individuals with social anxiety. We wanted to explore the VWM of socially anxious individuals using CDA to index the maintained representations in VWM. Consistent with prior reasoning, we proposed the hypotheses: HSA group would perform better than LSA group in the VWM task. For this, we set two kinds of memory load to observe this difference.

## Materials and Methods

### Participants

We used Gpower to calculate the sample size. Because studies of relationship between social anxiety and VWM were few, we set the parameters usually used in studies [effect size *f* = 0.25, *α* err prob = 0.05, power (1 − β err prob) = 0.8]; after calculation, the total sample size was 34. Individuals in the HSA group comprised 18 participants, who scored above 60 on the Liebowitz Social Anxiety Scale (LSAS, Liebowitz, 1987; He and Zhang, 2004; Lv et al., 2014). One participant with trial rejection rates over 25% was excluded from the sample. Finally, the HSA group consisted of 17 participants (male = 3, female = 14, age = 23.24 ± 3.49). The LSA group comprised 17 participants (male = 5, female = 12, age = 23.35 ± 2.29) and scored 35 or lower on the LSAS (Pan et al., 2006). The LSAS comprises of fear and an avoidance subscale. Every subscale includes 24 items, listing socially relevant situations. Participants were required to rate each item on a 4-point Likert scale ranging from 0 (none) to 3 (severely/usually). In our study, we calculated the total score by summing scores from both subscales, yielding a maximum score of 144. Higher scores were associated with higher levels of social anxiety. Internal consistency was excellent in this study (Cronbach’s *α* = 0.96). HSA and LSA groups had significant differences in LSAS (*M*_HSA_ = 79.88, SD_HSA_ = 15.05, *M*_LSA_ = 27.65, SD_LSA_ = 13.92, *F*_(1,32)_ = 110.41, *P* < 0.001, partial *η*^2^ = 0.78). In addition, the 13 items version of Beck Depression Inventory (BDI; Beck and Beck, 1972) was used to assess the level of depression of the participants. The scale consists of 13 items that are rated on a Likert scale ranging from 0 (no depressive mood) to 3 (severe depressive mood). Compared with the LSA group, participants in the HSA group reported higher levels of depression (*M*_HSA_ = 9.82, SD_HSA_ = 5.05, *M*_LSA_ = 5.12, SD_LSA_ = 6.23, *F*_(1,32)_ = 5.85, *P* = 0.021, partial *η*^2^ = 0.15).

All participants had right-handedness, normal or corrected-to-normal vision, and passed the Ishihara Color Test, which is a test for color blindness. They were paid 80 yuan for their participation. All participants provided written and informed consent before the experiment, and the procedures were approved by the Research Ethics Board of Capital Normal University.

### Stimuli

Each memory item was a colored square (1.23° × 1.23° of visual angle). The color was selected from a set of eight colors: red, orange, yellow, green, cyan, blue, purple, and pink [the color selected in this study is based on the color parameters in the CIE-LCH model, red (53, 60, 40), orange (53, 60, 70), yellow (53, 58, 102), green (53, 60, 140), cyan (55, 32, 105), blue (53, 52, 272), purple (53, 60, 320), and pink (53, 60, 360)]. All stimuli were presented in the symmetrical rectangular region (4.9° × 9.8°). There were two/four squares in each region with random positions, and the spacing between two squares was >2° (center to center). Stimuli were presented on a 17-in Sony CRT monitor (1,024 × 768 pixels, 100 Hz refresh rate), with a black background. Participants were seated in a comfortable chair in a dimly lit room at a 70-cm viewing distance.

### Procedure

**Figure 1:**
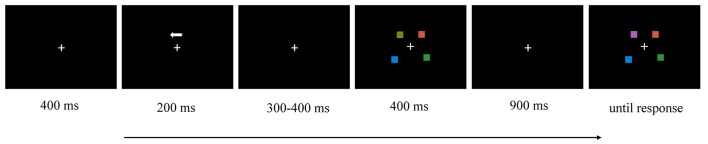
Illustration of the time course of a trial in this experiment (e.g., left hemifield, two memory items, change).

We adopted the change-detection task to measure an individual’s VWM capacity (see Figure 1). Each trial began with a fixation cross for 400 ms. Then, an arrow of 200 ms appeared as a cue, instructing the participant to remember the items in either the left or the right hemifield. Then, there was a random interval of 300–400 ms, followed by a memory array of 400 ms. After a 900-ms delay, the test array appeared, and the participant was demanded to determine whether the test array was identical or not from the memory array cued by the arrow. The color of one square in the test array was different from the corresponding item in the memory array in 50% of trials.

Two conditions were included: low-memory load (two memory items each hemifield) or high-memory load (four memory items each hemifield). Each block contained 72 trials, half of which changed and half of which did not. The experiment was divided into six blocks, and each condition contained three blocks.

### EEG Recording and Analysis

In this study, electroencephalograph (EEG) was recorded by Neuroscan ESI 64-channel recording system using the Ag/AgCl electrode cap of the international 10–20 extended electrode sites. EEG data were recorded by the left mastoid as reference, and re-referenced to the average of the left and right mastoids offline. The vertical electrooculogram (VEOG) generated from eye movements were monitored with two electrodes, placed approximately 1 cm above and below the left eye; horizontal eye movements were recorded from two electrodes placing approximately 1 cm beyond the outer edge of each eye. The electrode impedances were kept below 5 kΩ through the task. The EEG was amplified by SynAmps2 amplifiers with a bandpass of 0.1–125 Hz and sampled at 500 Hz. The recorded EEG data were filtered with low and high cutoffs of 0.05 and 40 Hz. EEG data exceeding ±75 μV were rejected. Trials with saccades (horizontal eye movements exceeding ±30 μV) and blinks (Fpz, ±60 μV) were discarded (Eimer and Kiss, 2010). Filtered data were segmented from 200 ms prior to the onset of the memory array until 1,400 ms, with a 200-ms baseline correction.

**Figure 2:**
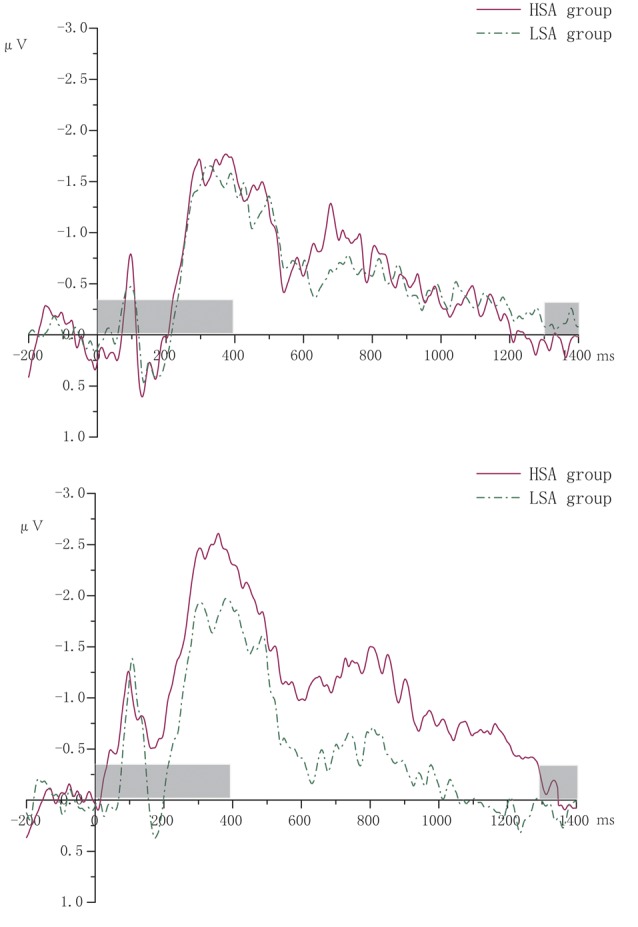
The grand averaged waveforms of contralateral delay activity (CDA) of high (HSA) and low socially anxious (LSA) groups. The left was for two memory items. The right was for four memory items. The gray-shaded area was the rendering phase of memory array or test array.

According to the literature (McCollough et al., 2007), ERPs focused on CDA (400–1,300 ms) with the four pairs of sites distributing in the posterior parietal lobe, occipital lobe, and temporal lobe (CP5/6, P7/8, P5/6, PO7/8). Contralateral waveforms were computed by averaging the activity recorded at right hemisphere electrode sites when participants were cued to remember the left side of the memory array with the activity recorded from the left hemisphere electrode sites when they were cued to remember the right side. CDA was measured as the difference in mean amplitude between the ipsilateral and contralateral waveforms. Figure 2 shows the grand averaged waveforms of CDA of HSA and LSA groups.

### Statistical Analysis

We used a formula *K* = *S* × (*H* − *FA*) to compute VWM capacity (Pashler, 1988; Cowan, 2001), where *K* is the VWM capacity, *S* is the size of the array (valid memory items), *H* is the hit rate, and *FA* is the false alarm rate. The larger the value of *K*, the stronger the VWM capacity.

*K* value and CDA amplitude data were entered into 2 × 2 mixed analysis of variance (ANOVA), respectively, with the group (HSA, LSA) as the between-subjects factor and the number of memory items (two/four) as a within-subjects factor. Appropriate Greenhouse–Geisser adjustments to the degrees of freedom were performed. Only significant (*P* < 0.05) interactions were further investigated for the analysis of simple effects with Bonferroni adjustments.

## Results

### Behavior Data

The ANOVA repeated measures performed on *K* value revealed a main effect for number (*F*_(1,32)_ = 49.72, *P* < 0.001, partial *η*^2^ = 0.61). The *K* value of four items (*M* = 2.28, SD = 0.08) was larger than that of two items (*M* = 1.78, SD = 0.02). No other significant main effect or interaction effect was observed (two items, *M*_HSA_ = 1.78, SD = 0.14, *M*_LSA_ = 1.77, SD = 0.08; four items, *M*_HSA_ = 2.27, SD = 0.53, *M*_LSA_ = 2.29, SD = 0.43).

### ERP Data

**Figure 3:**
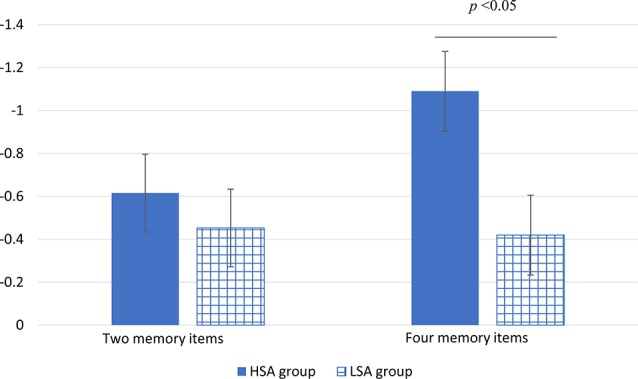
The mean amplitude of CDA in high (HSA) and LSA groups under different numbers of memory item conditions (the vertical bars extend and below the mean by one stand error).

Descriptiveness of CDA amplitude data recording from four pairs of electrodes is shown in Table 1. The ANOVA repeated measures conducted on CDA amplitudes showed a significant main effect for number (*F*_(1,32)_ = 4.54, *P* = 0.041, partial *η*^2^ = 0.12). The CDA amplitude of memorizing four items was larger (more negative) than that of memorizing two items. The main effect of group was marginally significant (*F*_(1,32)_ = 3.06 *P* = 0.09, partial *η*^2^ = 0.09). Compared with that of the LSA group, the CDA amplitude of the HSA group was larger. The interaction effect of group by number was significant (*F*_(1,32)_ = 6.01, *P* = 0.02, partial *η*^2^ = 0.16). Further simple effect analysis revealed that there was no significant difference in CDA amplitude between the HSA and LSA groups when the memory items were two. However, under the condition of four memory items, the HSA group showed significantly larger CDA amplitude than that of the LSA group (see Figure 3).

**Table 1:** Mean contralateral delay activity (CDA) amplitudes from four pairs of electrodes.

		CP5/6	P7/8	P5/6	PO7/8	Mean
HSA group	Two	−0.38 (0.95)	−0.59 (0.72)	−0.57 (0.89)	−0.93 (0.96)	−0.62 (0.79)
	Four	−0.80 (0.78)	−1.13 (0.80)	−1.17 (0.86)	−1.26 (0.80)	−1.09 (0.75)
LSA group	Two	−0.19 (1.09)	−0.35 (0.80)	−0.57 (0.83)	−0.71 (0.72)	−0.45 (0.70)
	Four	−0.09 (1.03)	−0.36 (0.70)	−0.39 (1.13)	−0.84 (0.79)	−0.42 (0. 79)

*HSA group, high socially anxious group; LSA group, low socially anxious group; Mean, mean amplitude of four pairs of sites*.

## Discussion

The current study was designed to explore the VWM of individuals with social anxiety. Behaviorally, the *K* value of the four memory items was higher than that of the two memory items. However, regardless of memory load, there was no difference in the *K* value between the HSA group and the LSA group.

In electrophysiology, we found that individuals with high social anxiety differ from individuals with low social anxiety in their VWM performance, which proved the hypothesis that we put forward. In particular, the CDA amplitude of HSA individuals in the VWM maintenance was significantly larger than that of LSA individuals under high memory load condition. However, there was no difference between the two groups under low memory load. Vogel et al. (2001) indicated that in memory array size of up to three items, participants showed a near-perfect performance but declined substantially for larger arrays. Accordingly, due to the ceiling effect, no difference in CDA amplitude was observed between the HSA and LSA groups when memory array was two items in the present study.

The electrophysiological results showed that high socially anxious (HSA) individuals exhibited better VWM performance than the low socially anxious (LSA) individuals in the four-item condition. This result is consistent with the findings of Moriya and Sugiura (2012). In their experiment 1, they asked participants to complete a change-detection task, which contained 4, 8, or 12 colored squares to be remembered, meanwhile measuring the participants’ trait social anxiety by the brief fear of negative evaluation scale. Moriya and Sugiura found a positive correlation between trait social anxiety and memory capacity. Part of the explanation for this result can be found in the attention control theory. Eysenck et al. (2007) mentioned that when people perceive themselves in a threatening situation and experience anxiety, it is ecological to allocate visual attention more widely to detect threats. Individuals with social anxiety are more sensitive to evaluation from others and fearful of negative evaluation than healthy people. In this regard, they may hold a large amount of information in VWM. In addition, Beilock and Carr (2005) indicated that only individuals with high working memory capacity were harmed by performance pressure, that is, anxiety consumed their working memory capacity available for an excellent performance.

In our result, we found a separation between response accuracy and CDA amplitude. In particular, the HSA and LSA groups showed no difference in behavioral performance under the condition of high memory load; by contrast, individuals with high social anxiety showed greater CDA amplitude than LSA individuals in electrophysiology. It should be noted that the response accuracy is not as direct as the ERP measures and is obtained after the offset of the test array. It reflects the processing of extraction stage of memory, while CDA reflects the storage stage. Under this line of reasoning, individuals with social anxiety may not extract all information they stored.

Generally, individuals with high working memory capacity have good skills for cognitive control, but when under high pressure, their performance is often impaired (Decaro et al., 2008). Social anxiety might play the role of pressure, moderating the relationship between VWM capacity and attentional control. Our results provide indirect support for the attentional control theory that individuals with anxiety have deficits in attentional control, which makes their bottom–up attention system enhanced and sensitive to environmental information. From the results, we can see that HSA people have greater VWM capacity, which would endow them with adequate capacity to process additional irrelevant information. Consequently, because of the deficit of inhibition, anxious individuals allocate resources to threat stimuli. To prove this, we can learn from the research paradigm of Lee et al. (2010) to further explore this issue in the future. In their study, they asked patients with Parkinson’s disease and education-matched control group to perform a change-detection task. There were three types of memory arrays in the task: 2-red–2-green trials, 2-red trials, and 4-red trials (each hemifield). The task required participants to remember the orientations of red rectangles within the cued half of the memory array while ignoring all green rectangles. With this task arrangement, they can explore the inhibition ability of participants.

The limitations of this study are the following. First of all, the participants in this study were nonclinical individuals with social anxiety, so the generalizability of the conclusions was limited. Second, we should measure the trait and state anxieties of participants, which are very more general form of anxiety, and may have an impact on our results. Third, we did not control for depression levels when we recruited participants. In order to exclude the influence of depression on the results, we conducted covariance analysis using depression as a covariable and found that the conclusion that individuals with social anxiety have better VWM was still supported. Lastly, the statistical power of the main effect of group on CDA is too small, which may be related with our small sample size. We will increase the sample size to improve our statistical power in future studies.

In summary, the current study explored the VWM of socially anxious individuals and found that individuals with social anxiety have large VWM resource to observe the environment around them, which implies that we can reduce social anxiety by training individuals with social anxiety to consciously focus on positive stimuli. It is of great significance for us to explore effective treatments of social anxiety. The results also provide evidence for processing efficiency theory, that is, when auxiliary cognitive resources are available, performance effectiveness is less likely to suffer.

## Data Availability Statement

The datasets generated for this study are available on request to the corresponding author.

## Ethics Statement

The studies involving human participants were reviewed and approved by the Research Ethics Board of Capital Normal University. The patients/participants provided their written informed consent to participate in this study.

## Author Contributions

JY analyzed the data and wrote the articles. NM and RC assisted in doing the experiment. QZ and LC directed the experiment.

## Conflict of Interest

The authors declare that the research was conducted in the absence of any commercial or financial relationships that could be construed as a potential conflict of interest.
